# Adaptive and Maladaptive DNA Breaks in Neuronal Physiology and Alzheimer’s Disease

**DOI:** 10.3390/ijms25147774

**Published:** 2024-07-16

**Authors:** Anysja Roberts, Russell H. Swerdlow, Ning Wang

**Affiliations:** 1 University of Kansas Alzheimer’s Disease Research Center, Kansas City, KS 66205, USArswerdlow@kumc.edu (R.H.S.); 2 Department of Neurology, University of Kansas Medical Center, Kansas City, KS 66160, USA; 3 Department of Biochemistry and Molecular Biology, University of Kansas Medical Center, Kansas City 66160, KS, USA; 4 Department of Cell Biology and Physiology, University of Kansas Medical Center, Kansas City, KS 66160, USA; 5 Institute for Reproductive and Developmental Sciences, University of Kansas Medical Center, Kansas City, KS 66160, USA

**Keywords:** DNA breaks, DNA damage, Alzheimer’s disease (AD), TOP2B, transcription

## Abstract

DNA strand breaks excessively accumulate in the brains of patients with Alzheimer’s disease (AD). While traditionally considered random, deleterious events, neuron activity itself induces DNA breaks, and these “adaptive” breaks help mediate synaptic plasticity and memory formation. Recent studies mapping the brain DNA break landscape reveal that despite a net increase in DNA breaks in ectopic genomic hotspots, adaptive DNA breaks around synaptic genes are lost in AD brains, and this is associated with transcriptomic dysregulation. Additionally, relationships exist between mitochondrial dysfunction, a hallmark of AD, and DNA damage, such that mitochondrial dysfunction may perturb adaptive DNA break formation, while DNA breaks may conversely impair mitochondrial function. A failure of DNA break physiology could, therefore, potentially contribute to AD pathogenesis.

## 1. Introduction

Postmitotic neurons are crucial elements of the nervous system that oversee essential body functions and complex cognitive processes such as memory formation. Despite being largely unrenewable, these neurons must carry out these vital roles while preserving their cellular and genomic integrity throughout many decades of life. As neurons age, their genomic integrity is reduced because DNA damage increases [1]. Alzheimer’s disease (AD) is a neurodegenerative disease with an increased risk in the older population and it is the most common cause of dementia [2,3]. Certain genotypes can also influence the risk of developing AD, with the *APOE4* allele having the highest impact [4,5]. Additionally, amyloid plaques composed mostly of amyloid β (Aβ) peptide, neurofibrillary tangles composed of hyperphosphorylated tau, inflammation, loss of synapse, and mitochondrial dysfunction are hallmarks of AD.

Mounting evidence now suggests a well-established link between the accumulation of DNA damage, indicative of genomic instability, and AD [6,7,8,9,10,11,12,13,14]. Meanwhile, increasing evidence has established that neuronal activity-dependent DNA breaks serve a functional role in experience-driven memory formation by promoting the expression of immediate early genes (IEGs) necessary for cellular and synaptic plasticity [15,16]. Thus, the term “adaptive” has been used to describe such roles of these DNA breaks in learning and memory [17]. This current review summarizes recent evidence, revealing the necessary role of neuronal activity-induced adaptive DNA breaks in experience-dependent memory formation. Meanwhile, we highlight recent studies suggesting that these adaptive DNA breaks may become “maladaptive” in AD. Additionally, given that the repair of DNA breaks is a highly energy-consuming process, we explore the connection between DNA breaks and mitochondrial defects in AD.

## 2. Adaptive Role of DNA Breaks in Learning and Memory

DNA breaks can occur in two main ways, depending on whether phosphodiester bonds break in one or both DNA strands, resulting in single-strand or double-strand breaks (SSBs and DSBs, respectively). Both types of DNA breaks are detected in neurons [15,18,19,20]. Homologous recombination (HR) is the preferred DSB repair pathway as it is relatively error-free. However, since HR needs to occur exclusively in the S or G2 phase of the cell cycle, and because neurons are postmitotic and non-replicating, the HR pathway is not available for neurons. Thus, neurons primarily rely on base excision repair (BER) and nucleotide excision repair (NER) pathways to repair SSBs, as well as non-homologous end joining (NHEJ) to repair DSBs [21]. The DNA damage and DNA damage response (DDR) have been reviewed recently [13,22].

Neuronal gene expression in the brain dynamically changes in response to neuronal activity. Notably, immediate-early genes (IEGs) such as *Egr-1*, *c-fos*, and *Arc* are rapidly and selectively upregulated in specific neurons within brain regions associated with learning and memory formation [23]. Therefore, IEG expression is crucial for mediating the plastic changes that underlie the formation of long-term memory. The detection of DNA breaks rapidly formed around IEGs provides mechanistic insight into how these genes are quickly expressed upon neuronal stimulation [17].

### 2.1. γH2AX as a Biomarker of DNA Damage

Many studies investigating DNA double-strand breaks (DSBs) rely on the detection of γH2AX (phosphorylated histone H2AX) as a key biomarker [24,25,26]. The nucleosome, which encompasses DNA and four core histones including H2A, undergoes modifications such as acetylation or phosphorylation in response to genomic alterations. H2AX, a subfamily of H2A, is a unique histone that has been evolutionarily conserved across species and is distinguished by its sensitivity to DNA breaks. Specifically, H2AX is rapidly phosphorylated at the serine 139 residue to become γH2AX by Ataxia telangiectasia mutated (ATM), Ataxia telangiectasia and a Rad3-related protein (ATR), and a DNA-dependent protein kinase catalytic subunit (DNA-PKcs). The formation of γH2AX in the presence of minimal DNA breaks, induced by low levels of ionizing radiation (IR), indicates its high sensitivity to DNA damage. Consequently, the formation of γH2AX in cells is considered one of the earliest responses to DNA breaks. Additionally, the number of γH2AX foci directly correlates with the amount of DNA breaks, making γH2AX a specific marker for DNA damage. However, γH2AX can spread up to 2 mega-bases away from the DNA break site, which hinders its use in precisely identifying the genomic positions of DNA breaks.

### 2.2. Evidence Establishing Neuronal Activity-Induced DNA Breaks

DNA breaks have historically been deemed cytotoxic to neurons, leading to cell senescence or apoptosis; however, recent studies have shown that neuronal DNA breaks form even in response to physiological brain activity [15]. An early study used N-methyl-D-aspartic acid (NMDA), which is an ionotropic glutamate receptor agonist, to mimic neuronal stimulation [27]. The data show that brief NMDA treatment induces DNA breaks without resulting in neuronal cell death. In this study, cells were treated with 15 μM or 50 μM of NMDA, and maximum levels of γH2Ax were seen after just ten minutes. Further, these γH2Ax levels returned to their corresponding control levels four hours after the treatment. These results suggest that γH2Ax activity is quickly and transiently induced. A second ionotropic glutamate receptor, α-amino-3-hydroxy-5-methyl-4-isoxazolepropionic acid (AMPA), was also used to mimic neuronal stimulation and understand how activation of non-NMDA receptors influences DNA breaks. Resembling NMDA treatment, 50 μM AMPA resulted in maximal γH2Ax levels after one hour. Accordingly, this study suggested for the first time that neuronal activity is an inducer of DNA breaks.

Following this finding, neuronal DNA breaks were shown to be involved in the memory formation process in vivo [20]. The study showed a transient increase in DNA breaks, as indicated by increased γH2Ax levels in brain regions associated with memories, including the dentate gyrus, when mice explored novel environments. This increase in DNA breaks was likely not caused by stress hormones because the mice were treated with corticosterone subcutaneously to maintain baseline corticosterone levels and the DNA break levels were similar to mice without corticosterone treatment. Significantly, the comet assay was utilized to verify that, alongside the emergence of γH2AX foci as a biomarker for detecting DNA breaks, DNA fragmentation was indeed increased in brain cells following exploration of a novel environment. After 24 h, γH2AX levels returned to the baseline level, supporting the transient formation and subsequent repair of DNA breaks. Additionally, the study exposed one eye in anesthetized mice to 15 min of visual stimulus and saw an upregulation of γH2AX levels in the corresponding visual cortex one hour later. Optogenetic stimulation was also utilized to activate neurons in the mouse striatum, resulting in the upregulation of γH2Ax levels specifically isolated within the striata. Together, these results indicate that physiological neuronal activity is the driving factor of increased DNA breaks. Interestingly, another group investigated specific forms of neuronal activity and found exposure to novel environments led to a greater increase in DNA breaks in comparison to mice that underwent more routine tasks [28]. These results suggest that neuronal activity alone does not induce DNA breaks; instead, DNA break formation is implicated in specific types of neuronal activities, particularly those involved in learning and memory formation.

**Figure 1:**
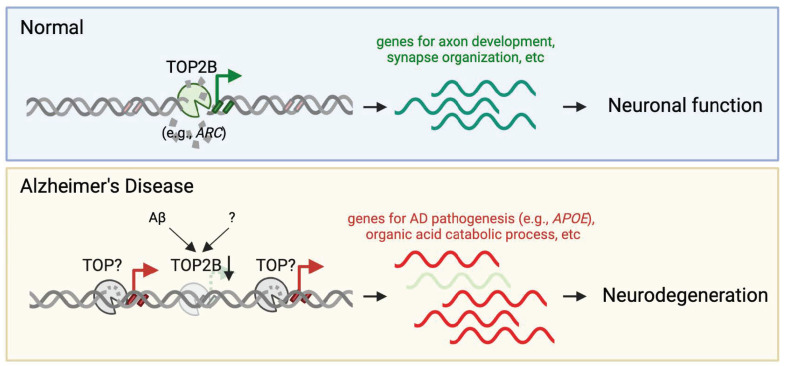
Schematic of DNA Break Dynamics in AD Brains. Despite a net increase in DNA breaks at ectopic genomic hotspots, there is a significant loss of TOP2B-catalyzed adaptive DNA breaks around synaptic genes. This loss of adaptive DNA breaks is correlated with transcriptomic dysregulation, indicating potential disruptions in gene expression related to synaptic function.

Why do neurons generate DNA breaks when stimulated? Immediate early genes (IEGs) provide a functional link between neuronal activity and DNA breaks (Figure 1, upper panel) [29]. IEG expression does not require protein synthesis and is rapidly induced by neuronal stimulation, highlighting their role as immediate responders to neural activity by swiftly translating external stimuli into intracellular responses. There are two categories of IEGs: the transcription factor IEGs, such as *Fos* and *Jun*, which indirectly promote synaptic plasticity by activating the transcription of genes needed for synaptic development and maturation, and the “effector” IEGs, which are directly involved in rapid response to neuronal activity and aid in promoting neuronal plasticity. A typical “effector” IEG is *Arc* (also known as *Arg3.1*), which encodes an activity-regulated cytoskeletal-associated protein that is critical for learning and memory formation [30,31,32]. Interestingly, Arc protein also mediates intercellular communication in the nervous system by forming virus-like capsids that carry *Arc* mRNA. Arc capsids are released from neurons in extracellular vesicles, thereby transferring *Arc* mRNA to new target cells [33].

The link between IEGs and DNA breaks generated by neuronal stimulation was identified using ChIP-Seq for γH2Ax, which profiled the genomic positions of DNA breaks in primary mouse neuronal cultures [15]. These neurons were treated with NMDA to mimic neuronal stimulation, and the study revealed enriched γH2AX within IEGs that are characterized by rapid expression during neuronal activity. Interestingly, the γH2AX peaks started near the transcription start site (TSS) and ended after the 3′ untranslated region, with the width of each peak corresponding to the length of the gene. Type II DNA topoisomerase β (Top2β) catalyzes the formation of these DNA breaks. This suggests that the DNA breaks specifically target IEGs in response to neuronal stimulation. Additionally, these DNA breaks around IEGs are repaired within two hours of initial stimulation, and the inhibition of DNA break repair results in prolonged IEG expression, illustrating the importance of DNA break repair (see Section 2.3). A follow-up study investigated the relationship between DNA break formation and IEG response in vivo by using contextual fear conditioning (CFC), which subjects mice to a novel environment with an adverse stimulus and, utilizing ChIP-Seq, revealed a rapid induction of DNA breaks within 30 min post-neuronal stimulus [34]. Comparing these data to RNA-Seq, they revealed that the DNA break sites corresponded to IEG activity after neuronal stimulus, further demonstrating that DNA breaks are critical for inducing IEG activity and achieving neuronal plasticity [34].

### 2.3. TOP2B—Catalyzing Activity-Dependent DNA Breaks for IEG Expression

Understanding that DNA breaks induce IEG activity, recent reports have demonstrated the functionality that DNA breaks employ in response to neuronal stimulation. One of the first modes of functionality revealed was transcription, which is catalyzed by TOP2B (Figure 1, upper panel) [15,16]. By catalyzing DNA break formation, TOP2B helps relax coiled DNA and alleviate tension to aid in replication or transcription [35]. In order to understand how TOP2B mediates DNA breaks, TOP2B was immunoprecipitated from neurons treated with NMDA, and the precipitate was incubated with supercoiled DNA, which showed TOP2B activity results in the relaxation of the coiled DNA and DNA breaks [15]. The study also found that treatment with NMDA produced a five-fold increase in IEG-bound TOP2B, suggesting TOP2B activity is induced by neuronal stimulation [15]. Further, the study reduced TOP2 activity by 50% and treated cells with NMDA, which reduced the number of γH2Ax within specific IEG, showing the importance of TOP2B in generating IEG activity within DNA breaks [15]. Following this finding, another study also utilized NMDA in addition to calcineurin inhibitors to determine how TOP2B activation occurs [16]. They found that neuronal stimulation generates an influx of calcium that activates phosphatase calcineurin, and calcineurin then activates TOP2B via dephosphorylation [16]. Together, these studies demonstrate the necessity of TOP2B activation in catalyzing activity-dependent DNA breaks and their importance in regulating neuronal activity and IEG transcription.

DNA breaks can also modulate gene expression by regulating transcriptional elongation. During transcriptional activation, DNA breaks occur to help facilitate gene transcription, resulting in γH2AX expression within actively transcribed genes [36]. Interestingly, γH2Ax activity increases within TSS and gene transcription regions when polymerase II (Pol II) is present [36]. Pol II activity is essential for transcriptional elongation; therefore, its association with DNA breaks implies they have an important role in transcriptional elongation [36]. Further, the inhibition of Cyclin-Dependent Kinase 9 (CDK9), which interrupts Pol II transcription, results in fewer DNA breaks within transcriptionally active genes [36]. These results suggest that DNA break activity directly correlates to Pol II activity and helps aid in transcription and elongation [36].

### 2.4. DNA Breaks and Memory Formation through Inflammatory Response

Most recently, it has been revealed that DNA breaks generate DNA fragments that induce inflammation to aid in memory formation [37]. Investigating the hippocampal cornu ammonis (CA1) and staining for γH2AX revealed that DNA breaks in neurons are associated with inflammatory signals. Further examination of signals associated with γH2AX, induced from CFC, showed that most differentially expressed genes were related to inflammation, suggesting that DNA breaks trigger an inflammatory response. Accordingly, the study identified that Tlr9 is activated by DNA fragments developed from activity-induced DNA breaks. Remarkably, the study found that inflammatory signaling through the Tlr9 receptor is necessary for memory formation, as neuron-specific knockdown of *Tlr9* impaired memory and reduced CFC-induced changes of gene expression. Therefore, these results suggest that activity-induced DNA breaks may be a beneficial and novel function in aiding memory formation by inducing an inflammatory response after neuronal stimulation.

### 2.5. Repair of Adaptive DNA Breaks

With the recurrent production of DNA breaks at hotspots, how do neurons repair these activity-dependent breaks? A recent study shed light on this important process while examining NPAS4, an activity-dependent transcription factor [38,39]. They found NPAS4 co-precipitates with the NuA4 chromatin-modifying complex in stimulated mouse hippocampal neurons. NPAS4 and NuA4 together promote neuronal activity-dependent transcription and DSB repair. Mapping DSB sites in the hippocampus showed a majority overlapped with high NPAS4 binding sites. Deletion of Npas4 or Tip60 caused DSB accumulation post-stimulation, indicating NPAS4-NuA4’s role in DSB repair. Higher mutation rates were found at NPAS4-bound sites in young mice but these sites resisted age-related mutational increases. This suggests NPAS4-mediated repair protects against age-associated mutations at sites regulating experience-driven behaviors.

## 3. Accumulation of DNA Damage in AD

Despite their necessity for neuronal functionality, studies have revealed increased levels of DNA breaks in AD [6,10,11,40]. Postmortem samples of AD and control patients were investigated and a two-fold increase in DNA breaks in the AD patients was found when compared to the control samples [40]. A later study used Klenow, Tunel, and Apostain to identify DNA breaks in the hippocampus of AD and control samples and also found that the most DNA breaks occurred in the AD samples [6]. Expanding on these findings, a more recent study used AD and mild cognitive impairment (MCI) samples to determine γH2AX activity specifically in neurons and astrocytes [10]. Both samples showed significantly higher DNA breaks when compared to the control samples in the neurons and astrocytes [10]. Surprisingly, they found similar levels of DNA breaks in both the AD and MCI samples, indicating that an increase in DNA breaks starts to occur with MCI [10]. This alteration in DNA breaks occurs across models as well, not just within human samples [11]. This correlates with the first mouse study investigating DNA breaks, where they saw an upregulation of γH2AX in human amyloid precursor protein (hAPP) mice [20]. Further, a study from 2021 saw increased DNA breaks in hippocampal samples from human and 5XFAD mice as well as in CHO 7PA2 Cells that are commonly used as AD models [11]. These studies reveal a significant accumulation of DNA breaks in AD and suggest that they may be detrimental with age.

The accumulation of DNA damage may result either from the attack on DNA by various internal and external factors, such as oxidative stress, radiation, and environmental toxins, or from a loss of repair mechanisms within the cell. In AD, both these conditions have been observed, indicating that the pathology of AD may be influenced by an increase in DNA damage due to these assaults and a concurrent decline in the efficiency of DNA repair processes [41]. One common cause of DNA damage in AD is the reactive oxygen species (ROS), which are highly reactive molecules that can cause significant damage to cellular components, including DNA, with an early study showing oxidative stress in AD patients leads to increased DNA damage, such as SSBs and DSBs [42]. More recent studies have also revealed increased ROS production within different AD models [41,43]. This increase in ROS production, and, consequently, increased DNA damage, describes one method by which DNA damage may accumulate in AD patients. Over time, the accumulation of such DNA damage can impair neuronal function and viability, contributing to the neurodegenerative process characteristic of AD. 8-Oxoguanine (8-oxoG), a major oxidative base lesion, is highly accumulated in AD brains during the pathogenic process [42]. Recently, several studies have shown that the mechanisms that mitigate the accumulation of 8-oxoG are defective in AD. For example, a reduction in histone deacetylase 1 (HDAC1) results in increased DNA damage [44]. HDAC1 is responsible for deacetylating 8-oxoGDNA glycosylase 1 (OGG1), which, in turn, removes the 8-oxoG DNA lesion [44]. However, when HDAC1 levels are downregulated, the OGG1 activity is reduced, leading to an upregulation of 8-oxoG DNA lesions [44]. This suggests that HDAC1 downregulation could be another reason for the increased 8-oxoG DNA lesions and, therefore, a cause for increased DNA breaks in AD patients. In addition, another recent study shows that both the levels of MTH1, which hydrolyzes 8-oxo-dGTP to 8-oxo-dGMP, thereby preventing 8-oxo-dG from incorporation into DNA, and OGG1 are significantly reduced in the brains of sporadic AD cases. When knocking out *Mth1* and *Ogg1* genes in 3xTg-AD, a commonly used genetic AD mouse model, MTH1 and OGG1 deficiency leads to a significant increase in 8-oxoG accumulation in nuclear genomes, accelerating microglial activation, neuronal degeneration, and cognitive deficit at 4–5 months of age [45].

DNA repair mechanisms may also be impaired in AD patients [8]. One such DNA repair factor is the MRE11/RAD50/NBS1 (MRN) complex, which utilizes non-homologous repair to mend DNA breaks [46]. Interestingly, it has been revealed that MRE11 and RAD50 expression are reduced in human hippocampal AD samples when compared to control samples [11]. However, these findings were not replicated in 5xFAD mice [11]. Another DNA repair factor is breast cancer factor 1 (BRCA1) [8]. BRCA1 helps repair DNA through homologous recombination [47], and a study using human amyloid precursor protein (hAPP) transgenic mice found reduced BRCA1 levels in the parietal cortex [8]. Following these results, they looked at BRCA1 levels in human samples and found them to be significantly reduced in the parietal cortex of both MCI and AD patients [8]. These findings are interesting because the study also investigated the consequence of BRCA1 knockdown and saw an increase in neuronal DNA breaks [8], indicating that the reduced BRCA1 levels in AD may contribute to an increase in neuronal DNA breaks. Together, these studies suggest that DNA break repair mechanisms are altered in AD patients, leading to an increase in DNA breaks in AD.

## 4. How Do the Positions of the DNA Damage Impact Neuronal Cell Function?

With the advancement of high-throughput genomic sequencing techniques, the contribution of DNA damage to DNA lesions, rearrangements, and mutations in neurons becomes increasingly apparent [18,19,48,49,50,51]. Notably, a recent study utilizing single-nucleus RNA-seq full-transcript analysis demonstrated that DNA damage plays a role in somatic mosaic gene fusions, particularly in excitatory neurons [14]. Similar alterations were observed in neurons derived from the CK-p25 mouse model of neurodegeneration. Neurons exhibiting an enrichment of DSBs also exhibited increased cohesin, alongside alterations in 3D genome organization, coinciding with transcriptional changes in genes related to synaptic function, neuronal development, and histones. Hence, the disruption of genome stability and 3D genome organization due to DNA breaks could significantly contribute to the progression of neurodegenerative diseases.

While these studies have focused on the role of DNA damage at the genomic level, a 2023 study mapped genome-wide DSB distributions in AD in autopsy brain tissue from AD and age-matched non-demented (ND) control individuals [52]. Nuclei extracted from frontal cortex tissue underwent Cleavage Under Targets and Release Using Nuclease (CUT&RUN) assaying to isolate γH2AX-occupied DNA, a proxy of DSBs, followed by high-throughput genomic sequencing. Aligning with previous findings indicating increased DNA breaks in AD brains, this study identified a significant increase in DSB numbers in AD brains. Intriguingly, it also revealed a distinct pattern of AD DSBs, appearing at “ectopic” genomic hotspots where ND DSBs were absent. Notably, an increase in DSBs was detected in the AD-associated genes, e.g., *APOE* and *APP*, suggesting a mechanism for their dysregulation in AD (Figure 1, lower panel). Furthermore, these ectopic DSBs correlated with AD-associated single-nucleotide polymorphisms (SNPs), increased chromatin accessibility, and elevated gene expression. Thus, this study suggests that AD DSBs not only increase in quantity but also manifest at ectopic genomic loci, potentially contributing to neuronal dysfunction and reprogramming in AD.

A subsequent investigation looked closer at the γH2AX CUT&RUN results, revealing that while DNA breaks occur at ectopic genomic loci, they are absent at “eutopic” genomic loci, where the adaptive nervous system genes crucial for neuronal function are located [53]. This observation was further corroborated by employing CUT&RUN for poly (ADP-ribose) (PAR), whereby DNA SSBs and DSBs activate poly (ADP-ribose) polymerases (PARPs) that conjugate PAR to adjacent proteins using nuclei from AD and ND autopsy brains. Despite the AD brains exhibiting more PAR peaks than the ND brains around genes enriched for catabolic processes, PAR peaks at the eutopic sites were notably diminished in AD brains, concomitant with reduced gene expression. An example of these genes is *ARC*, which is a classic IEG crucial for synaptic plasticity (Figure 1, lower panel) [30,31,32]. Moreover, the data indicate a significant decrease in TOP2B expression in AD brains. We consider that the reduction in TOP2B expression and loss of DNA breaks at nervous system genes may contribute to AD pathology. Conversely, AD pathology could instigate impaired neuronal function, leading to reduced TOP2B expression and the loss of DNA in nervous system genes. Interestingly, a study has shown that treatment of the Aβ_1-42_ peptide in cultured rat cerebellar granule neurons downregulates TOP2B expression at both mRNA and protein levels [54]. Nonetheless, the dual phenomena of increased DNA breaks at ectopic genomic loci and decreased DNA breaks at eutopic genomic loci likely synergize to induce maladaptive changes in AD neurons, with transcriptional dysregulation emerging as a possible mechanism.

While γH2AX and PAR CUT&RUN experiments have revealed the altered pattern of DNA breaks in AD brains, they serve as proxies. γH2AX often spread across the region flanking DSBs for several hundred kilobases, particularly in euchromatin (spread less efficiently in heterochromatin), which prevents the precise and unbiased mapping of DSBs by γH2AX. Thus, more recently developed technologies that directly label the DNA break sites, such as END-seq [55] or BLISS [56,57], will enhance the resolution of DNA breaks in AD brains.

It remains to be determined whether the downregulation of TOP2B expression and loss of eutopic DSBs are correlated or causal. Thus, further studies are necessary to establish whether TOP2B has a direct relationship with reduced functional DNA breaks. Beyond expression levels, TOP2B activity is triggered by calcineurin-induced dephosphorylation [16], and calcineurin signaling is known to be dysregulated in AD [58]. Thus, the measurement of TOP2B activity will indicate whether the catalytic activity of TOP2B is altered in AD. In this regard, a recent study examined TOP2B activity by trapping catalytically engaged TOP2B in covalent DNA cleavage complexes (TOP2Bccs) by treating cultured cells with etoposide (a TOP2 poison) and mapping their genome-wide distribution [59]. This revealed that TOP2B activity could vary relative to chromosome compartments but not loops and topologically associating domains (TADs). Additionally, various epigenetic changes are known to be present in AD [60], and alterations in the epigenetic landscape at TOP2B target sites may potentially change TOP2B recognition as well as its enzymatic activity in generating DSBs.

## 5. DNA Damage and Mitochondria Dysfunction

In addition to accumulating in nuclear DNA, DNA damage has also been detected in mitochondrial DNA (mtDNA), where it contributes to mitochondrial dysfunction [61]. Neurons require considerable amounts of energy. As mitochondria are essential for energy production and their efficiency declines with age [62], mitochondrial dysfunction is believed to represent a critical hallmark of neurodegenerative diseases, including Alzheimer’s disease [63,64,65].

Among its many detrimental effects, mitochondrial dysfunction likely interplays with DNA damage, with each exacerbating the other. Nicotinamide adenine dinucleotide (NAD^+^), a coenzyme in mitochondrial metabolism that facilitates the transfer of electrons during energy production (respiration), could link mitochondrial dysfunction and DNA breaks. Stressed mitochondria overproduce ROS, and one study found that an AD mouse model exhibited lower NAD^+^/NADH ratios along with increased ROS production [41]. When treated with nicotinamide riboside, a supplement known to upregulate NAD^+^ [66], these mice showed an increase in the NAD^+^/NADH ratio and a reduction in mitochondrial ROS production [41]. Interestingly, poly(ADP-ribose) polymerase 1 (PARP1), which is involved in the DNA repair process [67], is dependent on NAD^+^ and ATP [55]. Thus, reduced mitochondrial energy impairs PARP1 activity due to a lack of available energy (NAD^+^ and ATP), thus preventing proper DNA repair. Conversely, NAD^+^ activity activates sirtuin 1 (SIRT1) [68], which helps regulate glucogenesis and glycolysis and acts as an important regulator for the mitochondria with roles in biogenesis and mitophagy [69,70,71]. Interestingly, one study found that loss of PARP1 increases NAD^+^, which increases SIRT1 activity [68]. However, because PARP1 activity is increased by DNA breaks [67], NAD^+^ levels are reduced and SIRT1 activity is diminished, which maximizes mitochondrial dysfunction. These results suggest that hyperactive PARP1 could influence mitochondrial dysfunction. Collectively, these findings demonstrate that mitochondrial dysfunction in AD patients could contribute to increased DNA breaks, increased DNA breaks may contribute to mitochondrial dysfunction, or both.

## 6. Conclusions

DNA breaks are not only deleterious to the genome but also play a role in physiological functions in neurons. In Alzheimer’s disease (AD), DNA breaks accumulate at ectopic genomic locations where they are not normally detected, while adaptive DNA breaks that aid synaptic plasticity and memory formation are reduced. This raises important questions: What is the pattern of DNA breaks in unaffected brain regions in Alzheimer’s Disease (AD)? What is the contribution of single-strand breaks (SSBs) versus double-strand breaks (DSBs)? Are there specific alterations in DNA breaks associated with AD phenotypes? How does AD pathogenesis impact the formation of adaptive DNA breaks in response to stimuli? Does the loss of crucial adaptive DNA breaks mediate synaptic plasticity deficits and memory loss? Does the loss of adaptive DNA breaks indicate they were repaired, or do they lead to mutations? What mechanisms cause the reduction in adaptive DNA breaks? Together, these questions comprise the mechanistic gap in understanding how AD pathogenesis leads to synaptic and memory deficits through reduced adaptive DNA break formation. The information obtained could suggest DNA breaks at susceptible genomic loci and DNA topoisomerases (e.g., TOP2B) as novel diagnostic and therapeutic targets for AD.

## Data Availability

Not applicable.

## References

[1] Lu T., Pan Y., Kao S.Y., Li C., Kohane I., Chan J., Yankner B.A. (2004). Gene regulation and DNA damage in the ageing human brain. Nature.

[2] Kawas C., Gray S., Brookmeyer R., Fozard J., Zonderman A. (2000). Age-specific incidence rates of Alzheimer’s disease: The Baltimore Longitudinal Study of Aging. Neurology.

[3] Jellinger K.A. (2006). Clinicopathological analysis of dementia disorders in the elderly—An update. J. Alzheimers Dis..

[4] Farrer L.A., Cupples L.A., Haines J.L., Hyman B., Kukull W.A., Mayeux R., Myers R.H., Pericak-Vance M.A., Risch N., van Duijn C.M. (1997). Effects of age, sex, and ethnicity on the association between apolipoprotein E genotype and Alzheimer disease. A meta-analysis. APOE and Alzheimer Disease Meta Analysis Consortium. JAMA.

[5] Steinerman J.R., Irizarry M., Scarmeas N., Raju S., Brandt J., Albert M., Blacker D., Hyman B., Stern Y. (2008). Distinct pools of beta-amyloid in Alzheimer disease-affected brain: A clinicopathologic study. Arch. Neurol..

[6] Adamec E., Vonsattel J.P., Nixon R.A. (1999). DNA strand breaks in Alzheimer’s disease. Brain Res..

[7] Caldecott K.W. (2008). Single-strand break repair and genetic disease. Nat. Rev. Genet..

[8] Suberbielle E., Djukic B., Evans M., Kim D.H., Taneja P., Wang X., Finucane M., Knox J., Ho K., Devidze N. (2015). DNA repair factor BRCA1 depletion occurs in Alzheimer brains and impairs cognitive function in mice. Nat. Commun..

[9] McKinnon P.J. (2017). Genome integrity and disease prevention in the nervous system. Genes Dev..

[10] Shanbhag N.M., Evans M.D., Mao W., Nana A.L., Seeley W.W., Adame A., Rissman R.A., Masliah E., Mucke L. (2019). Early neuronal accumulation of DNA double strand breaks in Alzheimer’s disease. Acta Neuropathol. Commun..

[11] Thadathil N., Delotterie D.F., Xiao J., Hori R., McDonald M.P., Khan M.M. (2021). DNA Double-Strand Break Accumulation in Alzheimer’s Disease: Evidence from Experimental Models and Postmortem Human Brains. Mol. Neurobiol..

[12] Asada-Utsugi M., Uemura K., Ayaki T., Uemura M.T., Minamiyama S., Hikiami R., Morimura T., Shodai A., Ueki T., Takahashi R. (2022). Failure of DNA double-strand break repair by tau mediates Alzheimer’s disease pathology in vitro. Commun. Biol..

[13] Welch G., Tsai L.H. (2022). Mechanisms of DNA damage-mediated neurotoxicity in neurodegenerative disease. EMBO Rep..

[14] Dileep V., Boix C.A., Mathys H., Marco A., Welch G.M., Meharena H.S., Loon A., Jeloka R., Peng Z., Bennett D.A. (2023). Neuronal DNA double-strand breaks lead to genome structural variations and 3D genome disruption in neurodegeneration. Cell.

[15] Madabhushi R., Gao F., Pfenning A.R., Pan L., Yamakawa S., Seo J., Rueda R., Phan T.X., Yamakawa H., Pao P.C. (2015). Activity-Induced DNA Breaks Govern the Expression of Neuronal Early-Response Genes. Cell.

[16] Delint-Ramirez I., Konada L., Heady L., Rueda R., Jacome A.S.V., Marlin E., Marchioni C., Segev A., Kritskiy O., Yamakawa S. (2022). Calcineurin dephosphorylates topoisomerase IIbeta and regulates the formation of neuronal-activity-induced DNA breaks. Mol. Cell.

[17] Weber Boutros S., Unni V.K., Raber J. (2022). An Adaptive Role for DNA Double-Strand Breaks in Hippocampus-Dependent Learning and Memory. Int. J. Mol. Sci..

[18] Wu W., Hill S.E., Nathan W.J., Paiano J., Callen E., Wang D., Shinoda K., van Wietmarschen N., Colon-Mercado J.M., Zong D. (2021). Neuronal enhancers are hotspots for DNA single-strand break repair. Nature.

[19] Reid D.A., Reed P.J., Schlachetzki J.C.M., Nitulescu I., Chou G., Tsui E.C., Jones J.R., Chandran S., Lu A.T., McClain C.A. (2021). Incorporation of a nucleoside analog maps genome repair sites in postmitotic human neurons. Science.

[20] Suberbielle E., Sanchez P.E., Kravitz A.V., Wang X., Ho K., Eilertson K., Devidze N., Kreitzer A.C., Mucke L. (2013). Physiologic brain activity causes DNA double-strand breaks in neurons, with exacerbation by amyloid-beta. Nat. Neurosci..

[21] Neven J., Issayama L.K., Dewachter I., Wilson D.M. (2024). Genomic stress and impaired DNA repair in Alzheimer disease. DNA Repair..

[22] Wang H., Lautrup S., Caponio D., Zhang J., Fang E.F. (2021). DNA Damage-Induced Neurodegeneration in Accelerated Ageing and Alzheimer’s Disease. Int. J. Mol. Sci..

[23] West A.E., Greenberg M.E. (2011). Neuronal activity-regulated gene transcription in synapse development and cognitive function. Cold. Spring Harb. Perspect. Biol..

[24] Rogakou E.P., Pilch D.R., Orr A.H., Ivanova V.S., Bonner W.M. (1998). DNA double-stranded breaks induce histone H2AX phosphorylation on serine 139. J. Biol. Chem..

[25] Ward I.M., Chen J. (2001). Histone H2AX is phosphorylated in an ATR-dependent manner in response to replicational stress. J. Biol. Chem..

[26] Podhorecka M., Skladanowski A., Bozko P. (2010). H2AX Phosphorylation: Its Role in DNA Damage Response and Cancer Therapy. J. Nucleic Acids.

[27] Crowe S.L., Movsesyan V.A., Jorgensen T.J., Kondratyev A. (2006). Rapid phosphorylation of histone H2A.X following ionotropic glutamate receptor activation. Eur. J. Neurosci..

[28] Bellesi M., Bushey D., Chini M., Tononi G., Cirelli C. (2016). Contribution of sleep to the repair of neuronal DNA double-strand breaks: Evidence from flies and mice. Sci. Rep..

[29] Minatohara K., Akiyoshi M., Okuno H. (2015). Role of Immediate-Early Genes in Synaptic Plasticity and Neuronal Ensembles Underlying the Memory Trace. Front. Mol. Neurosci..

[30] Guzowski J.F., Lyford G.L., Stevenson G.D., Houston F.P., McGaugh J.L., Worley P.F., Barnes C.A. (2000). Inhibition of activity-dependent arc protein expression in the rat hippocampus impairs the maintenance of long-term potentiation and the consolidation of long-term memory. J. Neurosci..

[31] Guzowski J.F., McNaughton B.L., Barnes C.A., Worley P.F. (1999). Environment-specific expression of the immediate-early gene Arc in hippocampal neuronal ensembles. Nat. Neurosci..

[32] Bramham C.R., Worley P.F., Moore M.J., Guzowski J.F. (2008). The immediate early gene arc/arg3.1: Regulation, mechanisms, and function. J. Neurosci..

[33] Pastuzyn E.D., Day C.E., Kearns R.B., Kyrke-Smith M., Taibi A.V., McCormick J., Yoder N., Belnap D.M., Erlendsson S., Morado D.R. (2018). The Neuronal Gene Arc Encodes a Repurposed Retrotransposon Gag Protein that Mediates Intercellular RNA Transfer. Cell.

[34] Stott R.T., Kritsky O., Tsai L.H. (2021). Profiling DNA break sites and transcriptional changes in response to contextual fear learning. PLoS ONE.

[35] Bax B.D., Murshudov G., Maxwell A., Germe T. (2019). DNA Topoisomerase Inhibitors: Trapping a DNA-Cleaving Machine in Motion. J. Mol. Biol..

[36] Bunch H., Lawney B.P., Lin Y.F., Asaithamby A., Murshid A., Wang Y.E., Chen B.P., Calderwood S.K. (2015). Transcriptional elongation requires DNA break-induced signalling. Nat. Commun..

[37] Jovasevic V., Wood E.M., Cicvaric A., Zhang H., Petrovic Z., Carboncino A., Parker K.K., Bassett T.E., Moltesen M., Yamawaki N. (2024). Formation of memory assemblies through the DNA-sensing TLR9 pathway. Nature.

[38] Lin Y., Bloodgood B.L., Hauser J.L., Lapan A.D., Koon A.C., Kim T.K., Hu L.S., Malik A.N., Greenberg M.E. (2008). Activity-dependent regulation of inhibitory synapse development by Npas4. Nature.

[39] Pollina E.A., Gilliam D.T., Landau A.T., Lin C., Pajarillo N., Davis C.P., Harmin D.A., Yap E.L., Vogel I.R., Griffith E.C. (2023). A NPAS4-NuA4 complex couples synaptic activity to DNA repair. Nature.

[40] Mullaart E., Boerrigter M.E., Ravid R., Swaab D.F., Vijg J. (1990). Increased Levels of DNA Breaks in Cerebral Cortex of Alzheimer’s Disease Patients. Neurobiol. Aging.

[41] Turunc Bayrakdar E., Uyanikgil Y., Kanit L., Koylu E., Yalcin A. (2014). Nicotinamide treatment reduces the levels of oxidative stress, apoptosis, and PARP-1 activity in Abeta(1-42)-induced rat model of Alzheimer’s disease. Free Radic. Res..

[42] Gabbita S.P., Lovell M.A., Markesbery W.R. (1998). Increased nuclear DNA oxidation in the brain in Alzheimer’s disease. J. Neurochem..

[43] Sheehan J.P., Swerdlow R.H., Miller S.W., Davis R.E., Parks J.K., Parker W.D., Tuttle J.B. (1997). Calcium Homeostasis and Reactive Oxygen Species Production in Cells Transformed by Mitochondria from Individuals with Sporadic Alzheimer’s Disease. J. Neurosci..

[44] Pao P.C., Patnaik D., Watson L.A., Gao F., Pan L., Wang J., Adaikkan C., Penney J., Cam H.P., Huang W.C. (2020). HDAC1 modulates OGG1-initiated oxidative DNA damage repair in the aging brain and Alzheimer’s disease. Nat. Commun..

[45] Oka S., Leon J., Sakumi K., Abolhassani N., Sheng Z., Tsuchimoto D., LaFerla F.M., Nakabeppu Y. (2021). MTH1 and OGG1 maintain a low level of 8-oxoguanine in Alzheimer’s brain, and prevent the progression of Alzheimer’s pathogenesis. Sci. Rep..

[46] Dinkelmann M., Spehalski E., Stoneham T., Buis J., Wu Y., Sekiguchi J.M., Ferguson D.O. (2009). Multiple functions of MRN in end-joining pathways during isotype class switching. Nat. Struct. Mol. Biol..

[47] Moynahan M.E., Chiu J.W., Koller B.H., Jasin M. (1999). Brca1 Controls Homology-Directed DNA Repair. Mol. Cell.

[48] Lodato M.A., Woodworth M.B., Lee S., Evrony G.D., Mehta B.K., Karger A., Lee S., Chittenden T.W., D’Gama A.M., Cai X. (2015). Somatic mutation in single human neurons tracks developmental and transcriptional history. Science.

[49] Wei P.C., Chang A.N., Kao J., Du Z., Meyers R.M., Alt F.W., Schwer B. (2016). Long Neural Genes Harbor Recurrent DNA Break Clusters in Neural Stem/Progenitor Cells. Cell.

[50] McConnell M.J., Moran J.V., Abyzov A., Akbarian S., Bae T., Cortes-Ciriano I., Erwin J.A., Fasching L., Flasch D.A., Freed D. (2017). Intersection of diverse neuronal genomes and neuropsychiatric disease: The Brain Somatic Mosaicism Network. Science.

[51] Rodin R.E., Dou Y., Kwon M., Sherman M.A., D’Gama A.M., Doan R.N., Rento L.M., Girskis K.M., Bohrson C.L., Kim S.N. (2021). The landscape of somatic mutation in cerebral cortex of autistic and neurotypical individuals revealed by ultra-deep whole-genome sequencing. Nat. Neurosci..

[52] Zhang X., Liu Y., Huang M., Gunewardena S., Haeri M., Swerdlow R.H., Wang N. (2023). Landscape of Double-Stranded DNA Breaks in Postmortem Brains from Alzheimer’s Disease and Non-Demented Individuals. J. Alzheimers Dis..

[53] Zhang X., Haeri M., Swerdlow R.H., Wang N. (2024). Loss of Adaptive DNA Breaks in Alzheimer’s Disease Brains. J. Alzheimers Dis..

[54] Terzioglu-Usak S., Negis Y., Karabulut D.S., Zaim M., Isik S. (2017). Cellular Model of Alzheimer’s Disease: Abeta1-42 Peptide Induces Amyloid Deposition and a Decrease in Topo Isomerase IIbeta and Nurr1 Expression. Curr. Alzheimer Res..

[55] Canela A., Sridharan S., Sciascia N., Tubbs A., Meltzer P., Sleckman B.P., Nussenzweig A. (2016). DNA Breaks and End Resection Measured Genome-wide by End Sequencing. Mol. Cell.

[56] Yan W.X., Mirzazadeh R., Garnerone S., Scott D., Schneider M.W., Kallas T., Custodio J., Wernersson E., Li Y., Gao L. (2017). BLISS is a versatile and quantitative method for genome-wide profiling of DNA double-strand breaks. Nat. Commun..

[57] Bouwman B.A.M., Agostini F., Garnerone S., Petrosino G., Gothe H.J., Sayols S., Moor A.E., Itzkovitz S., Bienko M., Roukos V. (2020). Genome-wide detection of DNA double-strand breaks by in-suspension BLISS. Nat. Protoc..

[58] Norris C.M. (2018). Calcineurin: Directing the damage in Alzheimer disease: An Editorial for ‘Neuronal calcineurin transcriptional targets parallel changes observed in Alzheimer disease brain’ on page 24. J. Neurochem..

[59] Segev A., Heady L., Crewe M., Madabhushi R. (2024). Mapping catalytically engaged TOP2B in neurons reveals the principles of topoisomerase action within the genome. Cell Rep..

[60] Nikolac Perkovic M., Videtic Paska A., Konjevod M., Kouter K., Svob Strac D., Nedic Erjavec G., Pivac N. (2021). Epigenetics of Alzheimer’s Disease. Biomolecules.

[61] Tran M., Reddy P.H. (2020). Defective Autophagy and Mitophagy in Aging and Alzheimer’s Disease. Front Neurosci.

[62] Gomes A.P., Price N.L., Ling A.J., Moslehi J.J., Montgomery M.K., Rajman L., White J.P., Teodoro J.S., Wrann C.D., Hubbard B.P. (2013). Declining NAD(+) induces a pseudohypoxic state disrupting nuclear-mitochondrial communication during aging. Cell.

[63] Wang W., Zhao F., Ma X., Perry G., Zhu X. (2020). Mitochondria dysfunction in the pathogenesis of Alzheimer’s disease: Recent advances. Mol. Neurodegener..

[64] Swerdlow R.H. (2023). The Alzheimer’s Disease Mitochondrial Cascade Hypothesis: A Current Overview. J. Alzheimers Dis..

[65] Hirai K., Aliev G., Nunomura A., Fujioka H., Russell R.L., Atwood C.S., Johnson A.B., Kress Y., Vinters H.V., Tabaton M. (2001). Mitochondrial abnormalities in Alzheimer’s disease. J. Neurosci..

[66] Murata M.M., Kong X., Moncada E., Chen Y., Imamura H., Wang P., Berns M.W., Yokomori K., Digman M.A. (2019). NAD+ consumption by PARP1 in response to DNA damage triggers metabolic shift critical for damaged cell survival. Mol. Biol. Cell.

[67] Ahel D., Horejsi Z., Wiechens N., Polo S.E., Garcia-Wilson E., Ahel I., Flynn H., Skehel M., West S.C., Jackson S.P. (2009). Poly(ADP-ribose)-dependent regulation of DNA repair by the chromatin remodeling enzyme ALC1. Science.

[68] Bai P., Canto C., Oudart H., Brunyanszki A., Cen Y., Thomas C., Yamamoto H., Huber A., Kiss B., Houtkooper R.H. (2011). PARP-1 inhibition increases mitochondrial metabolism through SIRT1 activation. Cell Metab..

[69] Elesela S., Morris S.B., Narayanan S., Kumar S., Lombard D.B., Lukacs N.W. (2020). Sirtuin 1 regulates mitochondrial function and immune homeostasis in respiratory syncytial virus infected dendritic cells. PLoS Pathog..

[70] Rodgers J.T., Lerin C., Haas W., Gygi S.P., Spiegelman B.M., Puigserver P. (2005). Nutrient control of glucose homeostasis through a complex of PGC-1alpha and SIRT1. Nature.

[71] Yao Z.Q., Zhang X., Zhen Y., He X.Y., Zhao S., Li X.F., Yang B., Gao F., Guo F.Y., Fu L. (2018). A novel small-molecule activator of Sirtuin-1 induces autophagic cell death/mitophagy as a potential therapeutic strategy in glioblastoma. Cell Death Dis..

